# Supplementary material for: Effects of Pyrroloquinoline Quinone on Lipid Metabolism and Anti-Oxidative Capacity in a High-Fat-Diet Metabolic Dysfunction-Associated Fatty Liver Disease Chick Model
Source: Int J Mol Sci. 2021 Feb 1;22(3):1458. doi: 10.3390/ijms22031458 (PMC7867196; doi:10.3390/ijms22031458)
Supplement: Supplementary file 1 [file ijms-22-01458-s001.pdf]

Supplemental materials

Table S1. Composition and nutrition content of experimental diets, as fed basis.

Items	Control diet	High-energy low-protein diet
Ingredients, %		
Corn	62. 92	64. 37
Soybean meal	26. 23	18. 50
Limestone	8. 89	9. 00
Chicken fat	-	6. 00
Premix[1]	1. 96	2. 13
Total	100. 00	100. 00
Nutrient levels[2]		
ME, MJ/kg	11. 03	12. 75
Crude protein, %	16. 20	13. 00
Ca, %	3. 44	3. 50
Total P, %	0. 54	0. 50
Available P, %	0. 36	0. 34
Lys, %	0. 79	0. 74
Met, %	0. 36	0. 35

[1] The premix provided the followings per kg of diets: VA 8,000 IU, VD 2,200 IU, VE 30 IU, VK 2 mg, thiamine 1 mg, riboflavin 5.5 mg, calcium pantothenate 13 mg, niacin 36 mg, pyri-doxine 8 mg, biotin 0.5 mg, folic acid 0.5 mg, VB12 0.02 mg, Mn 65 mg, I 1 mg, Fe 60 mg, Cu 8 mg, Zn 66 mg, Se 0.3 mg, CaH_3PO_4 130 g, and NaCl 30 g.

[2] Nutrient levels were calculated values.

Table S2. The primers used for qRT-PCR assays.

Gene name	Primer sequence (5'~3')	Annealing temperature (℃)	Product size (bp)
PGC-1α	F: TTCGGGCTGAACTGAATAAG<br>R: CACTGTCATCAAAGAGACCATC	60	171
NRF-1	F: TGATGGCACTGTCCCTC<br>R: CCAGTTCTGCTCCACCTCTC	59	142
NRF-2	F: ATCACGAGCCCTGAAACCAA<br>R: GGCTGCAAAATGCTGGAAAA	59	143
TFAM	F: CAGGATGATAAGGTTCGGTA<br>R: TGGGCAGTGTCAGTCTTCTT	58	125
UCP-1	F: CGAACTGCCCAATTAGCCAA<br>R: TAATTCCAACACCACCTGCC	60	190
mt-ATP8	F: ATCCTCACTACTGTCATCTTAAC<br>R: AGTATGATGGAGAATCATGG	59	133
β-actin	F: ATCCGGACCCTCCATTGTC<br>R: AGCCATGCCAATCTCGTCTT	58	120

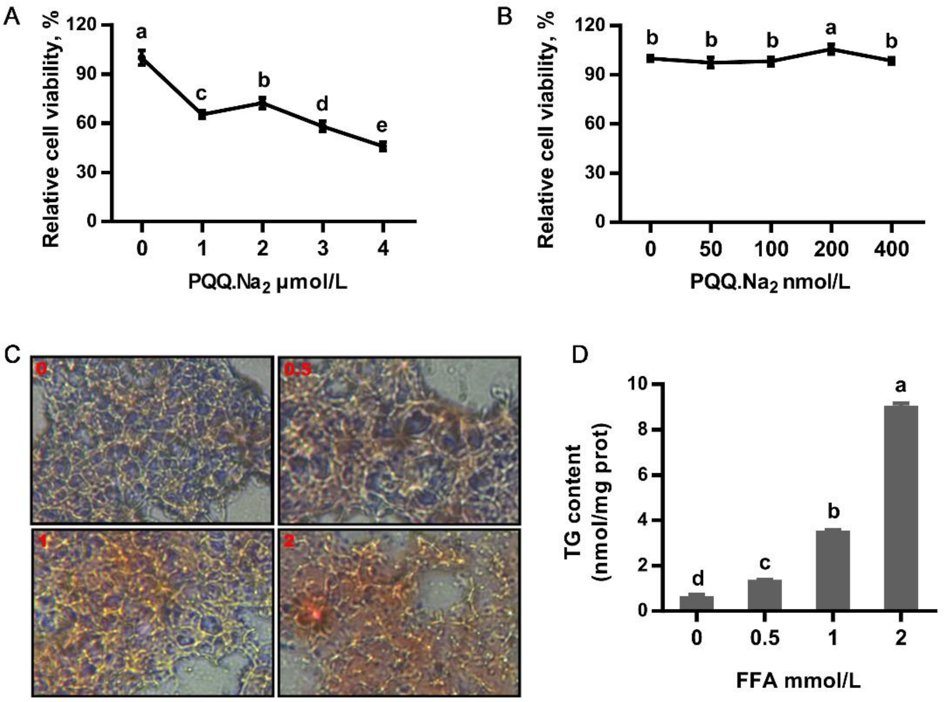

Figure S1. PQQ.Na2 and FFA treatments on primary hepatocytes of hens. (A-B) Relative cell viability of primary hepatocytes treated with Pyrroloquinoline quinone disodium (PQQ.Na2). (C) Oil Red O staining of primary hepatocytes treated with free fatty acid (FFA). (D) The triglyceride (TG) content of cells in C. Line nodes or bars without same small letters mean significant difference ($P < 0.05$).
